# Supplementary material for: Automated cell type annotation and exploration of single-cell signaling dynamics using mass cytometry
Source: iScience. 2024 Jun 12;27(7):110261. doi: 10.1016/j.isci.2024.110261 (PMC11253510; doi:10.1016/j.isci.2024.110261)
Supplement: Document S1. Figures S1–S15 and Tables S1 and S2 [file mmc1.pdf]

iScience, Volume *27*

Supplemental information

Automated cell type annotation and exploration of single-cell signaling dynamics using mass cytometry

Dimitrios Kleftogiannis, Sonia Gavasso, Benedicte Sjo Tislevoll, Nisha van der Meer, Inga K.F. Motzfeldt, Monica Hellesøy, Stein-Erik Gullaksen, Emmanuel Griessinger, Oda Fagerholt, Andrea Lenartova, Yngvar Fløisand, Jan Jacob Schuringa, Bjørn Tore Gjertsen, and Inge Jonassen

SUPPLEMENTARY FIGURES AND TABLES

Supplementary Figure 1: Overview of the annotated dataset used as reference, related to STAR Methods. a) Original UMAP representation of the annotated reference dataset showing 31 cell types with more than 300 cells available; b) Barplot showing the relative abundances of 31 cell types found in the reference dataset; c) Original UMAP representation of the annotated reference dataset with overlaid expression of selected cell type defining markers; d) Force-directed graph generated using the Scaffold map approach. Nodes represent all cell types found in the reference dataset (43 in total without filtering of 300 cells), and edges indicate similarity between the cell types. A cosine similarity cut-off of 0.75 has been used to remove edges between nodes.

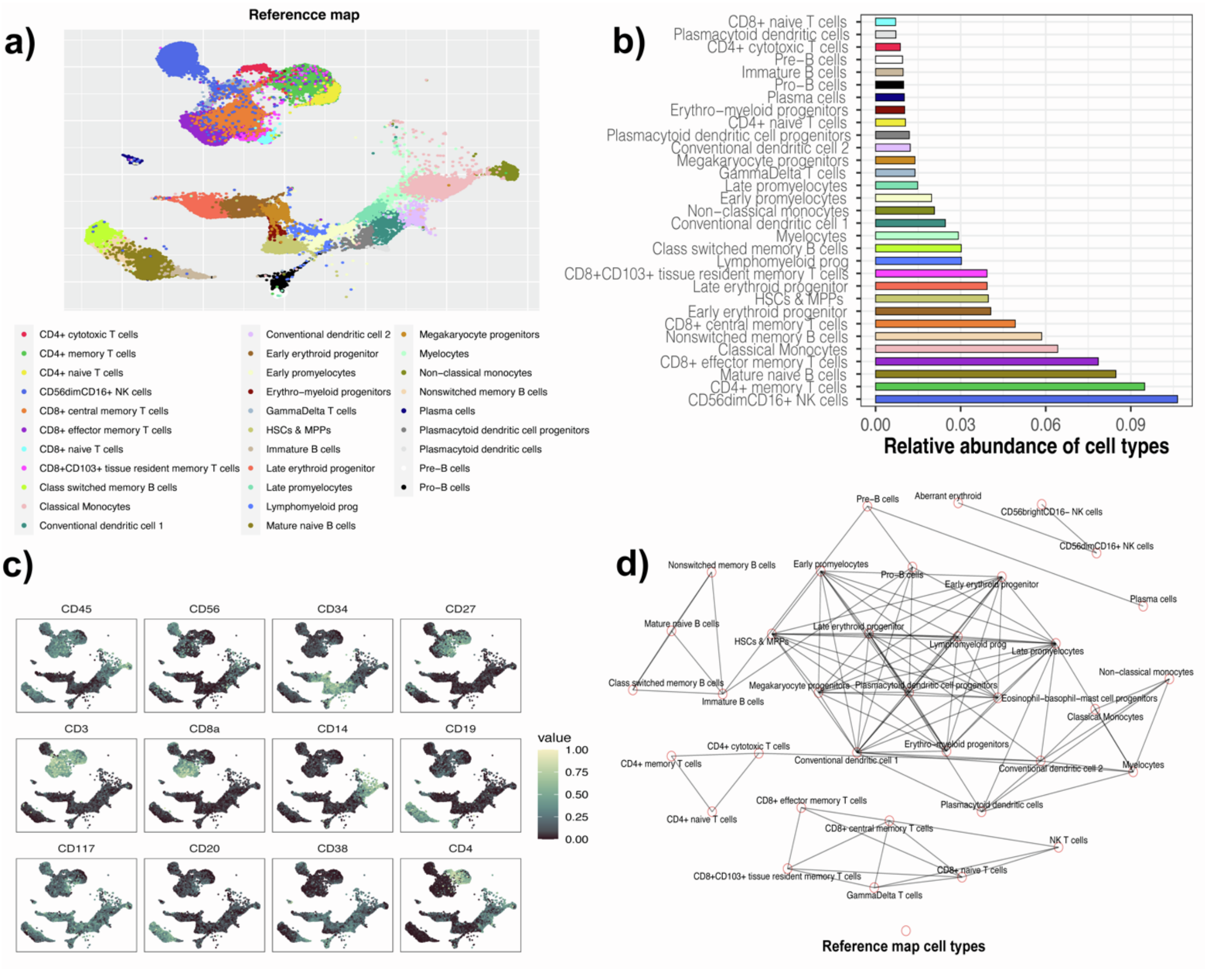

Supplementary Figure 2: Performance evaluation using a self-consistency test, related to Figure 1.

Results summarising the classification performance of Scaffold, CyAnno, LDA and KNN with K=3 and KNN with K=10, across 31 cell types from the Reference dataset, when split 50-50 is applied to generate disjoint sets for training and testing.

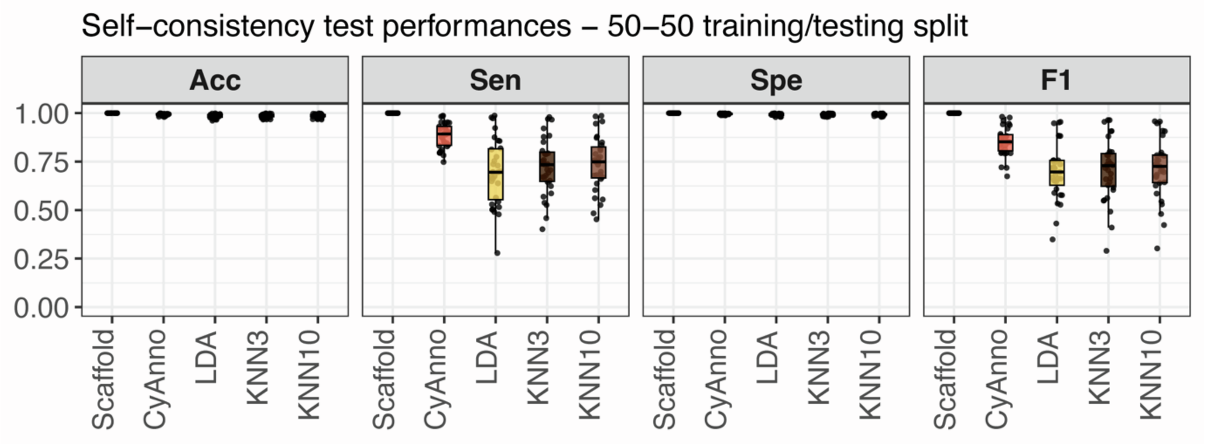

Supplementary Figure 3: Overview of mass cytometry datasets used as benchmarks for cell type annotation, related to Figure 1. Barplot showing the relative abundance of cell types found in: a) AML_benchmark; b) BMMC_benchmark; and c) PANORAMA_benchmark.

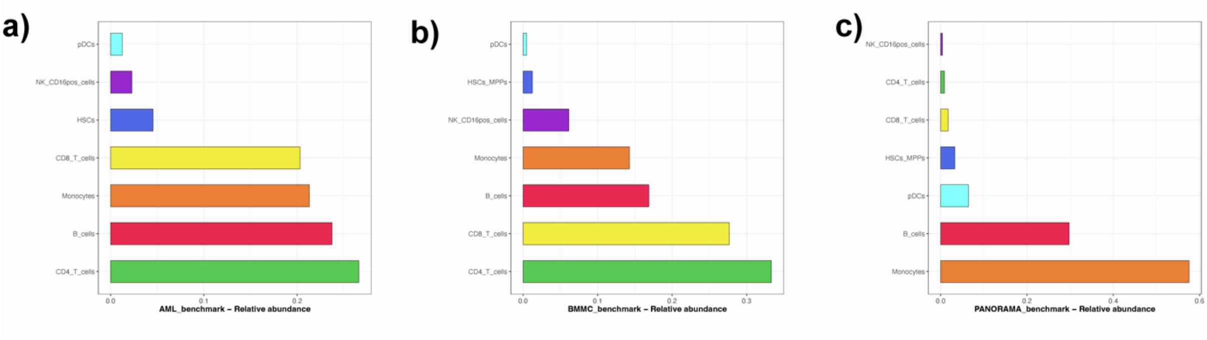

Supplementary Figure 4: Inspecting marker expression profiles of the benchmark datasets, related to Supplementary Figure 3. Ridge plots showing the expression profiles of selected cell type defining markers for seven cell types found in: a) AML_benchmark; b) BMMC_benchmark; and c) PANORAMA_benchmark. For visualization purposes, the expression values were scaled between 0 and 1 using low (1%) and high (99%) percentiles as boundaries. The x-axis shows the density of the scaled expression.

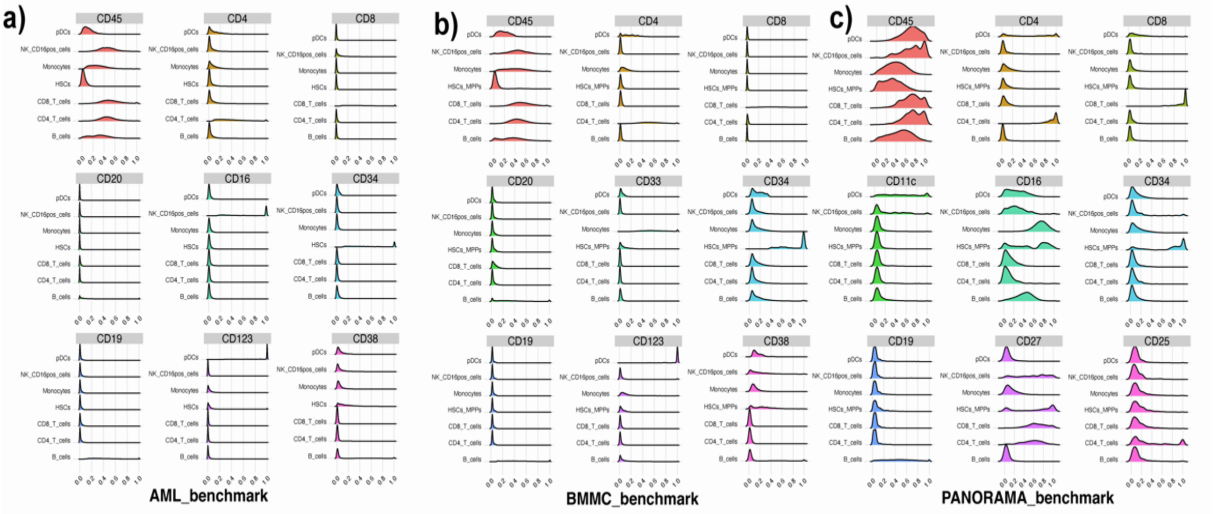

Supplementary Figure 5: UMAP projections to the reference dataset, related to Figure 2. a) UMAP visualisation of the reference dataset constructed using a smaller set of 'backbone' markers namely: CD11b, CD8a, CD33, CD34, CD3, CD123, CD56, CD14, CD117, CD38, CD4, CD16 ,CD20, CD45, CD7 ; b) UMAP projection of the healthy control dataset to the reference dataset constructed using the same set of 'backbone' markers; c) UMAP projection of the leukemia cohort to the reference dataset constructed using the same set of 'backbone' markers.

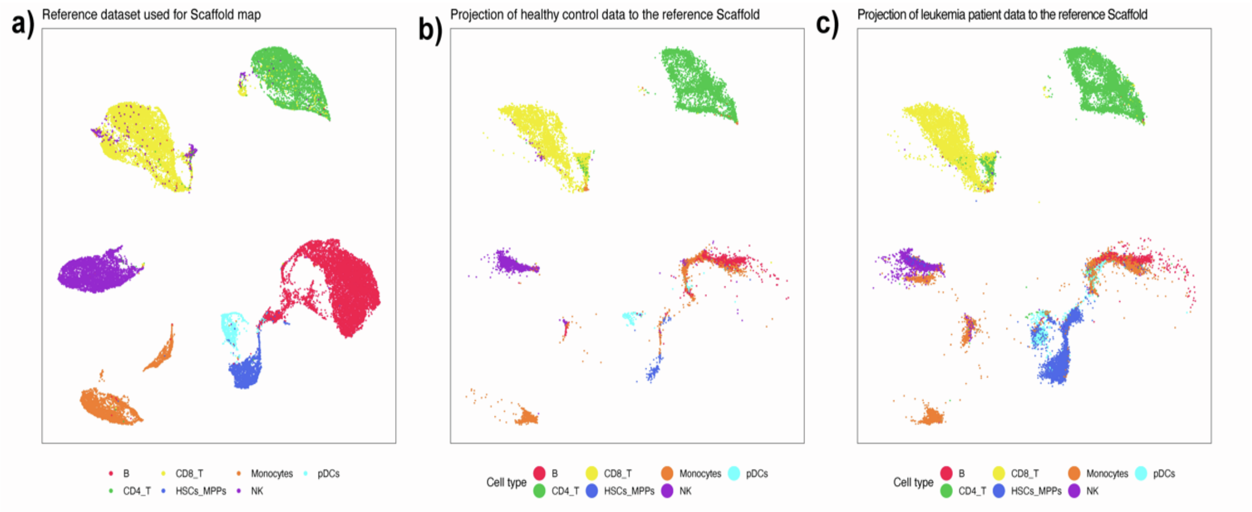

Supplementary Figure 6: Inspecting marker expression profiles of the annotated AML_benchmark, related to Figure 1. a) UMAP representation of the annotated dataset with overlaid expression of selected cell type defining markers; b) Ridge plots showing the expression profiles of selected cell type defining markers for the predicted cell types.

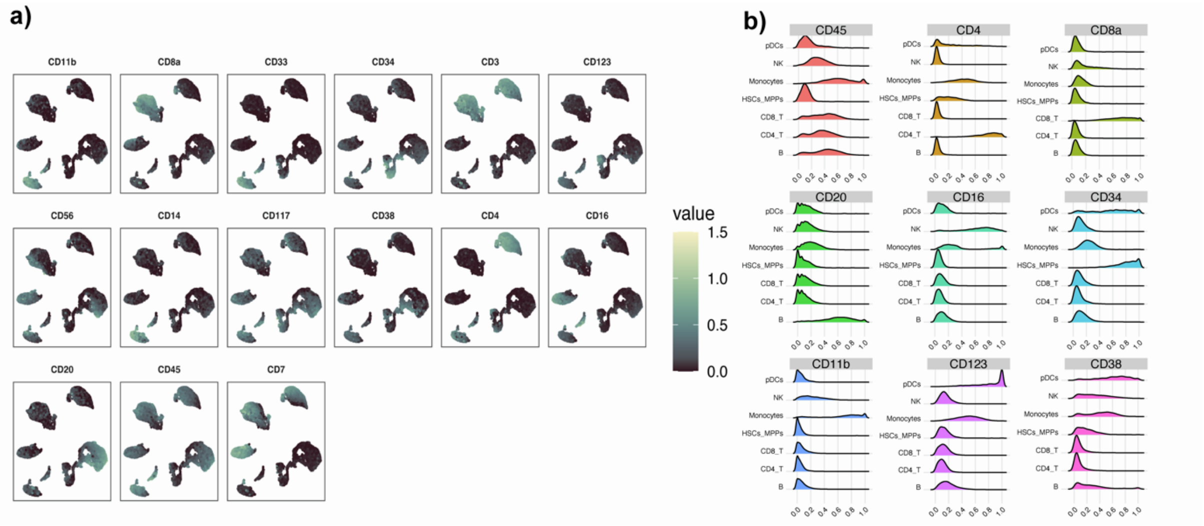

Supplementary Figure 7: Inspecting marker expression profiles of the annotated BMMC_benchmark, related to Figure 1. a) UMAP representation of the annotated dataset with overlaid expression of selected cell type defining markers; b) Ridge plots showing the expression profiles of selected cell type defining markers for the predicted cell types.

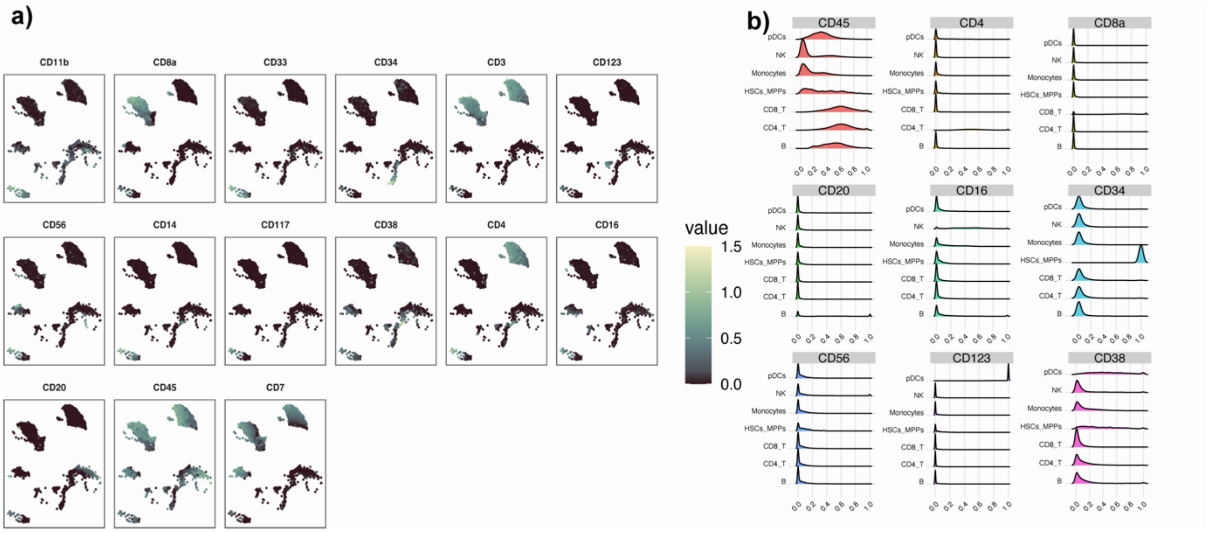

Supplementary Figure 8: Inspecting marker expression profiles of the annotated PANORAMA_benchmark, related to Figure 1. a) UMAP representation of the annotated dataset with overlaid expression of selected cell type defining markers; b) Ridge plots showing the expression profiles of selected cell type defining markers for the predicted cell types found in PANORAMA_benchmark .

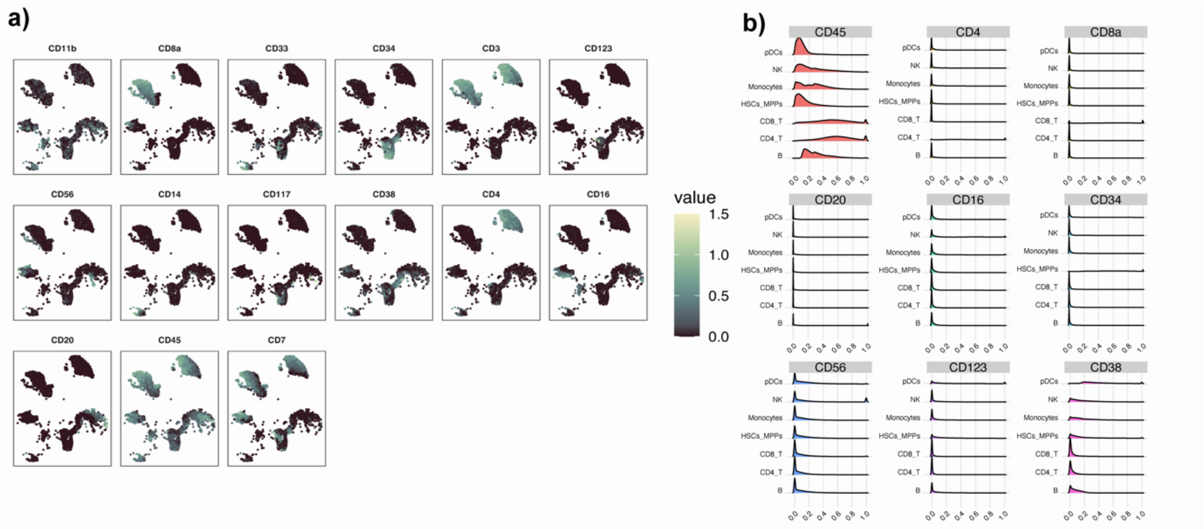

Supplementary Figure 9: Visualising Schoenfeld residuals for multivariate Cox proportional hazard modelling, related to Figure 2.

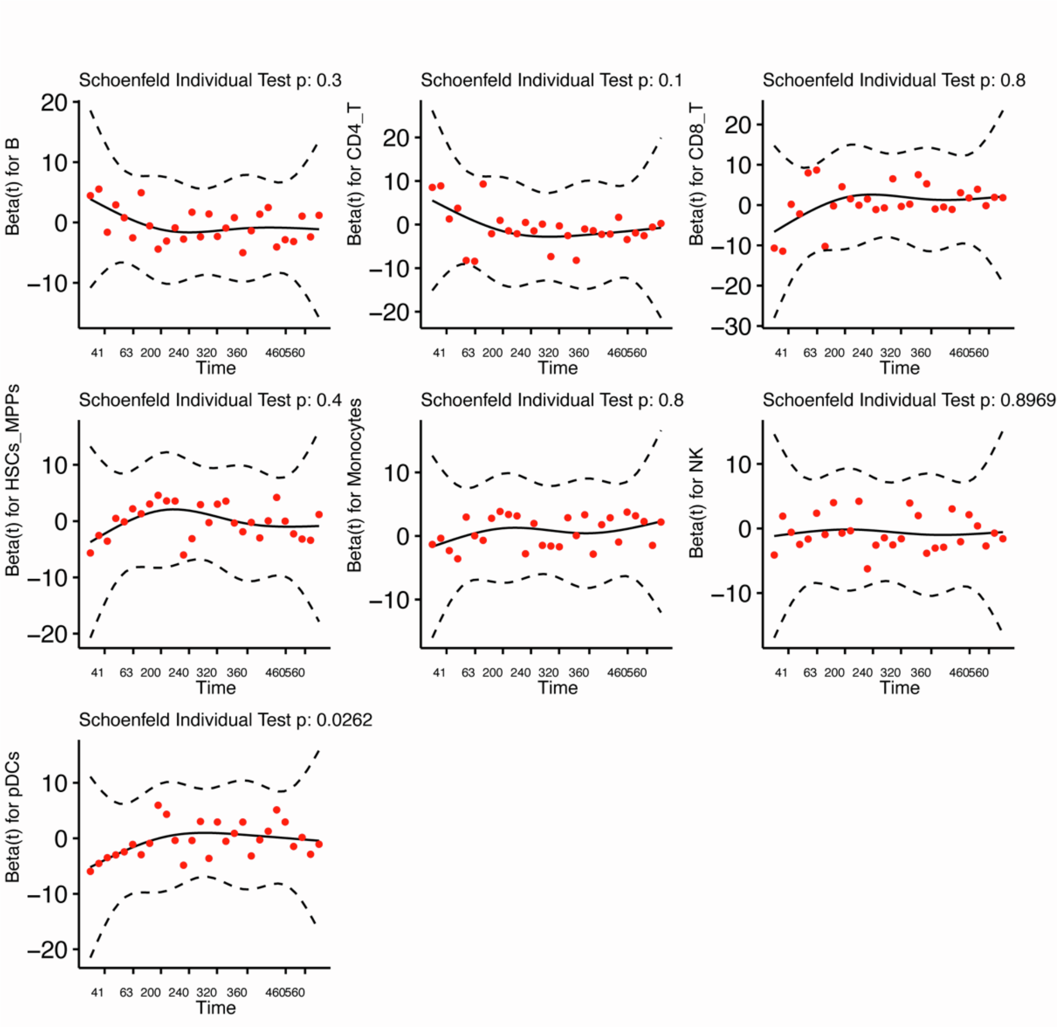

Supplementary Figure 10: Comparing cellular abundance distributions of leukemia patients stratified by genetic information, related to Figure 2. a) Boxplots showing the cellular abundance of patients with karyotypes classified as normal, complex, and chromosomally altered; b) Boxplots showing the cellular abundance of patients with FLT3-ITD mutated and FLT3-ITD wild type status. Statistically significant differences in a) were tested using anova with TukeyHSD correction, and in b) using Wilcoxon rank sum test. The levels of significance are: * for p<0.05, ** for p<0.01 and *** for p<0.001.

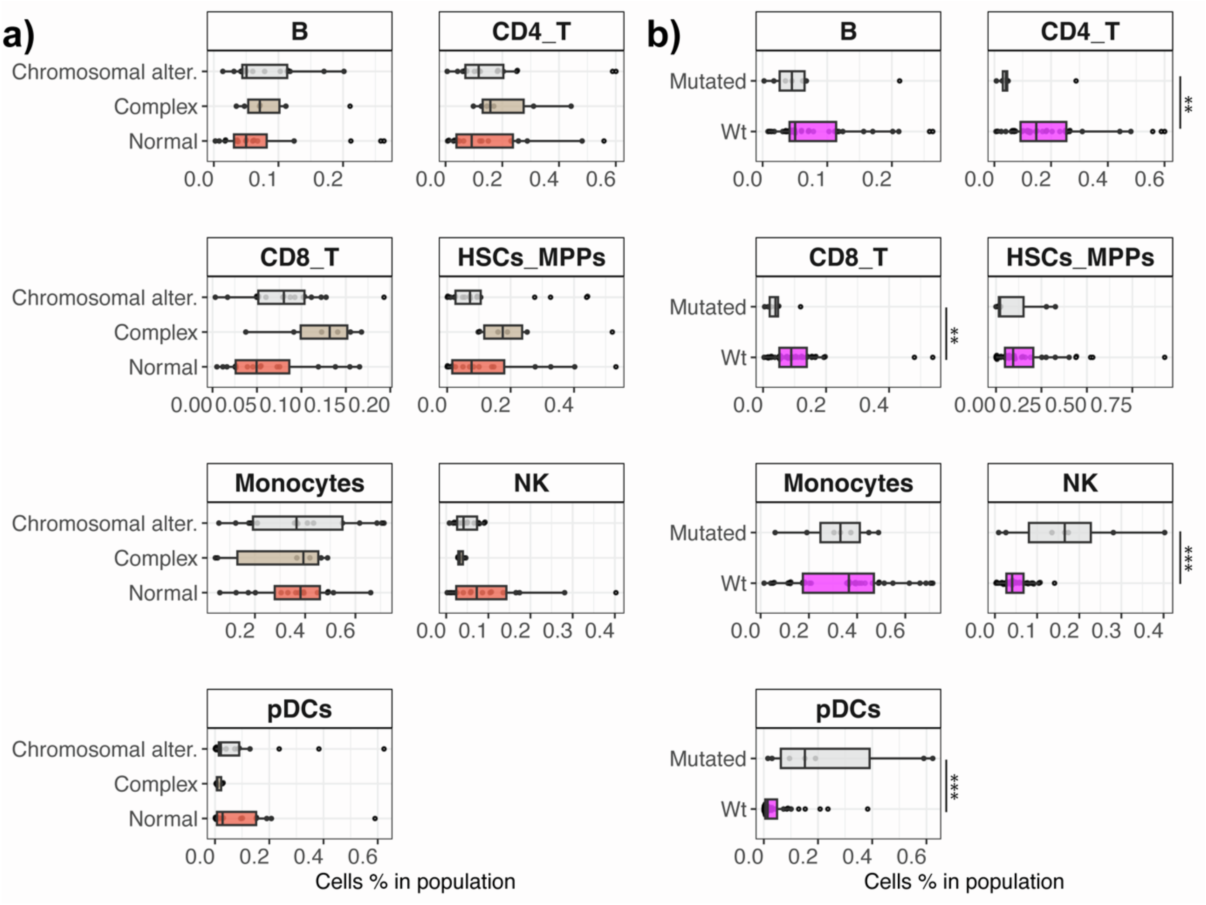

Supplementary Figure 11: The workflow of the developed bioinformatics framework, related to STAR Methods. Part 1 illustrates the cell type annotation method using the Scaffold map approach, and Part 2 illustrates the ML-based prediction of survival. Image was created using bioRender.

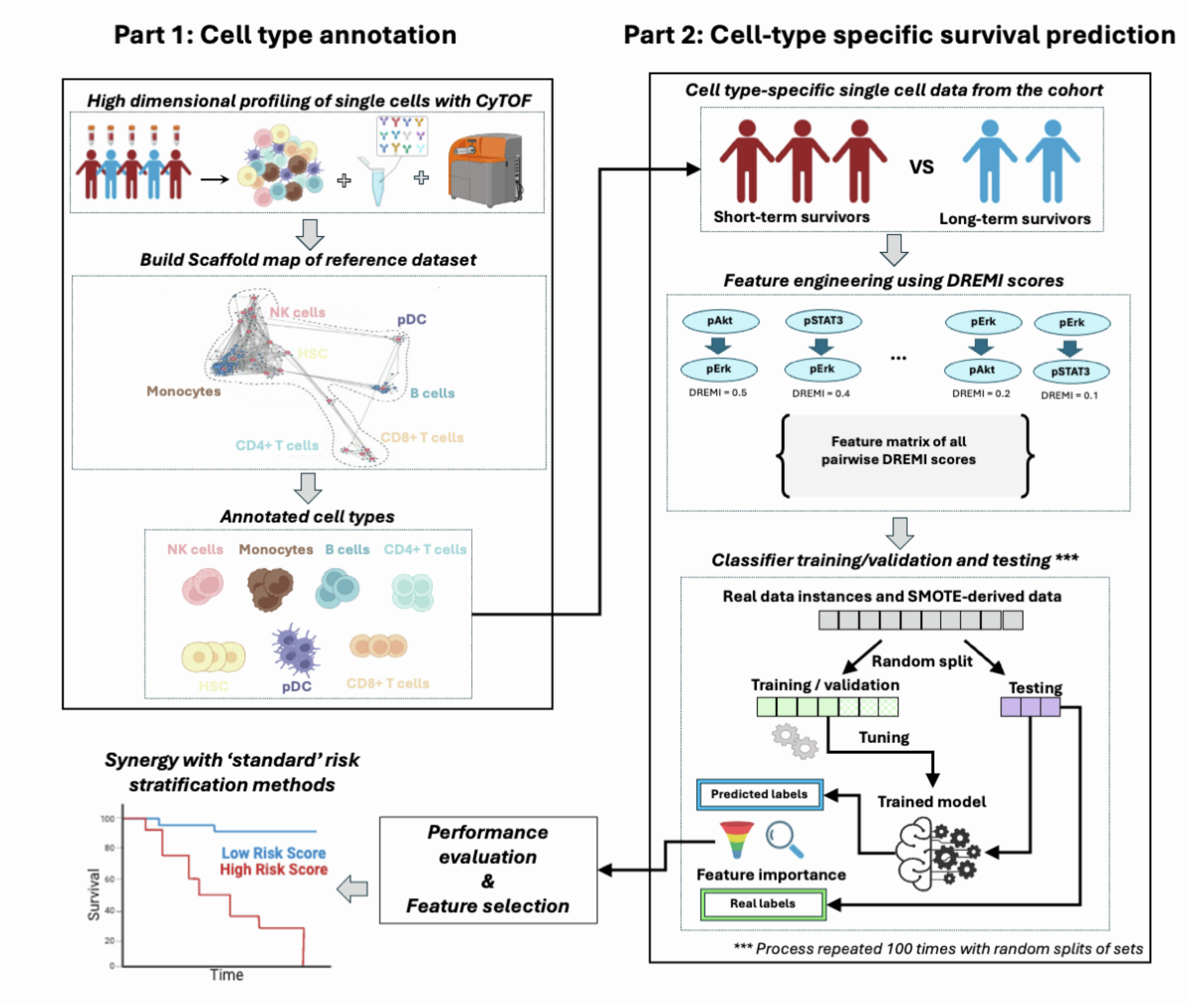

Supplementary Figure 12: Performance evaluation of different ML-based methods for predicting survival using as input features median expression values of phosphoproteins, related to Figure 3. For all experiments 50-50 random split of training/validation and testing sets is performed and average classification performance of XGBoost, LASSO and Ridge regression for discriminating STS from LTS patients is shown for: a) the data from the original case study cohort; b) SMOTE-derived synthetic data with ratio 1:1 between the classes; b) SMOTE-derived synthetic data with ratio 1:2 between the classes; c) SMOTE-derived synthetic data with ratio 1:3 between the classes. The performance is assessed using mean Sensitivity (SEN), mean Specificity (SPE), mean F1 and mean Area Under Curve (AUC) of 100 executions.

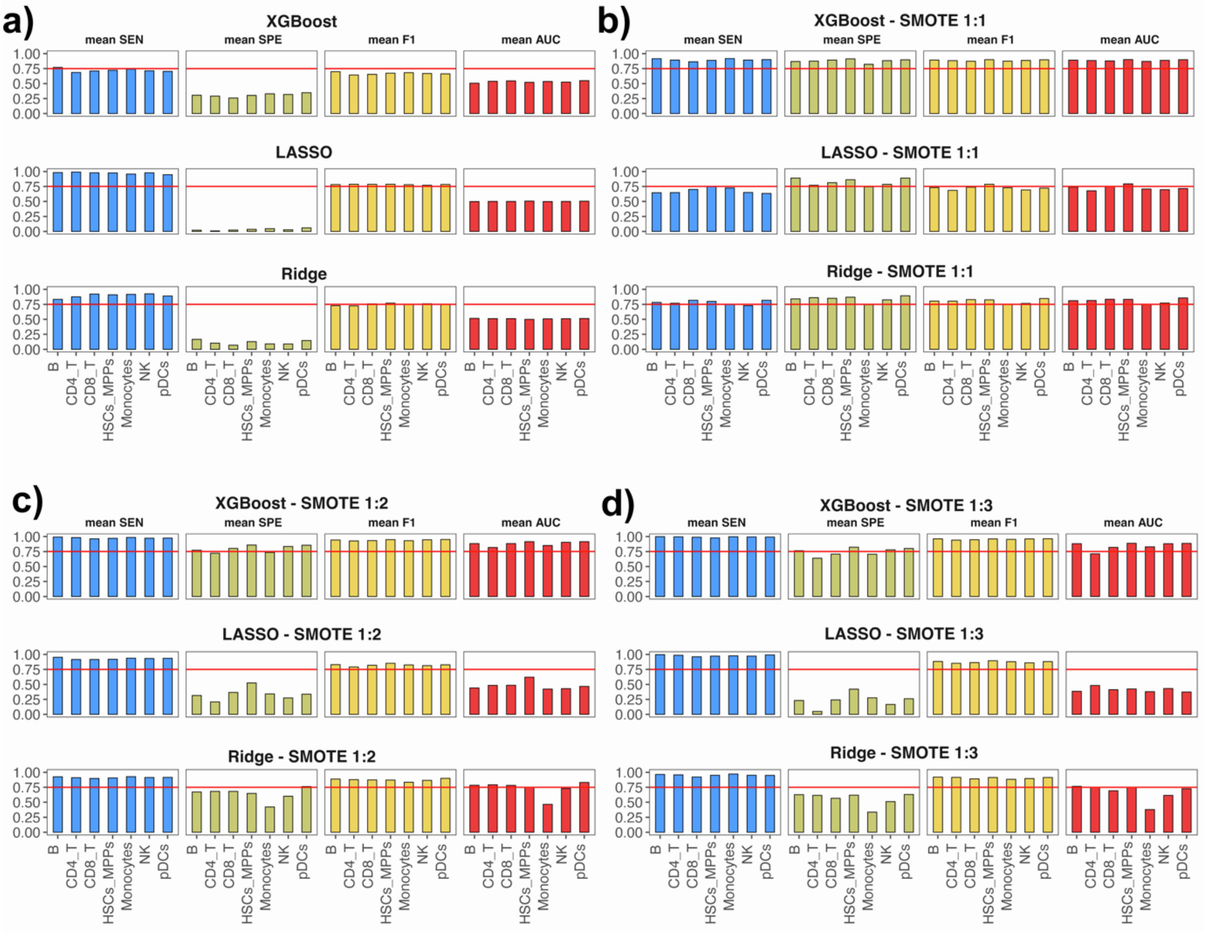

Supplementary Figure 13: Visualising the distribution of the most important DREMI scores learnt from the XGBoost models, related to Figure 4. a) Boxplots showing B cell derived DREMI scores of pP38-pSTAT3 for STS and LTS patients; b) Boxplots showing CD4_T cell derived DREMI scores of pSTAT3-pCREB for STS and LTS patients; c) Boxplots showing CD8_T cell derived DREMI scores of pCREB- pSTAT3 for STS and LTS patients; d) Boxplots showing HSC_MPP cell derived DREMI scores of pSTAT3-pErk for STS and LTS patients; e) Boxplots showing Monocyte derived DREMI scores of pErk-pSTAT5 for STS and LTS patients; f) Boxplots showing NK cell derived DREMI scores of pS6-pSTAT5 for STS and LTS patients; g) Boxplots showing pDCs derived DREMI scores of p4EBP1-Casp3 for STS and LTS patients.

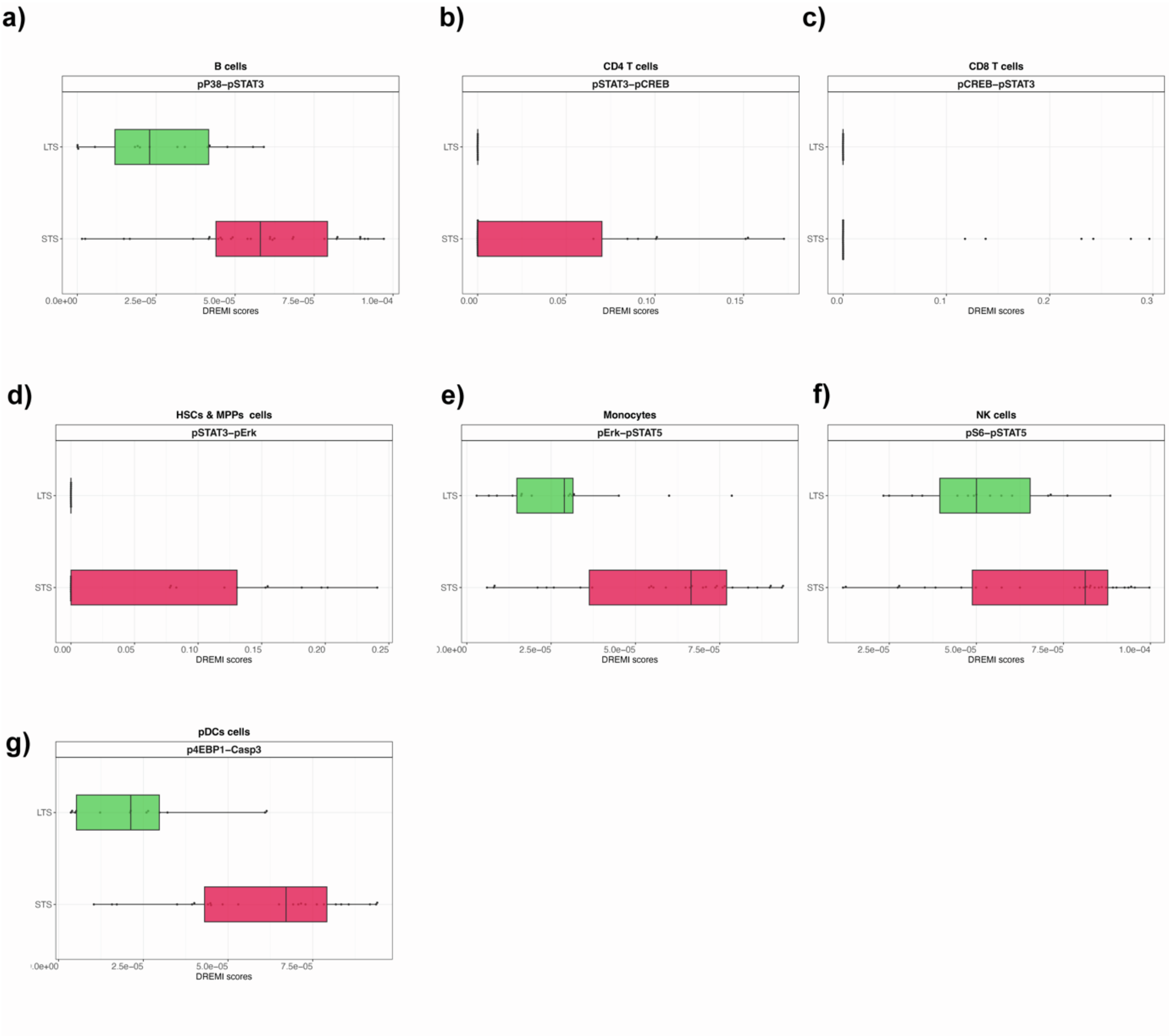

Supplementary Figure 14: Independent survival analysis using the most important DREMI scores learnt from the XGBoost models, related to Supplementary Figure 13. a) Survival probability of patients with pP38-pSTAT3 low vs high DREMI scores for B cells; b) Survival probability of patients with pSTAT3-pCREB low vs high DREMI scores for CD4_T cells; c) Survival probability of patients with pCREB-pSTAT3 low vs high DREMI scores for CD8_T cells; d) Survival probability of patients with pSTAT3-pErk pSTAT3 low vs high DREMI scores for HSC_MPP cells; e) Survival probability of patients with pErk-pSTAT5 low vs high DREMI scores for Monocytes; f) Survival probability of patients with pS6-pSTAT5 low vs high DREMI scores for NK cells; g) Survival probability of patients with p4EBP1-Casp3 low vs high DREMI scores for pDCs; h) Cox proportional hazards modelling using as input the most important cell type-specific DREMI scores learnt from the ML models. For subplots a-g univariate survival analysis is performed using Kaplan-Meier method.

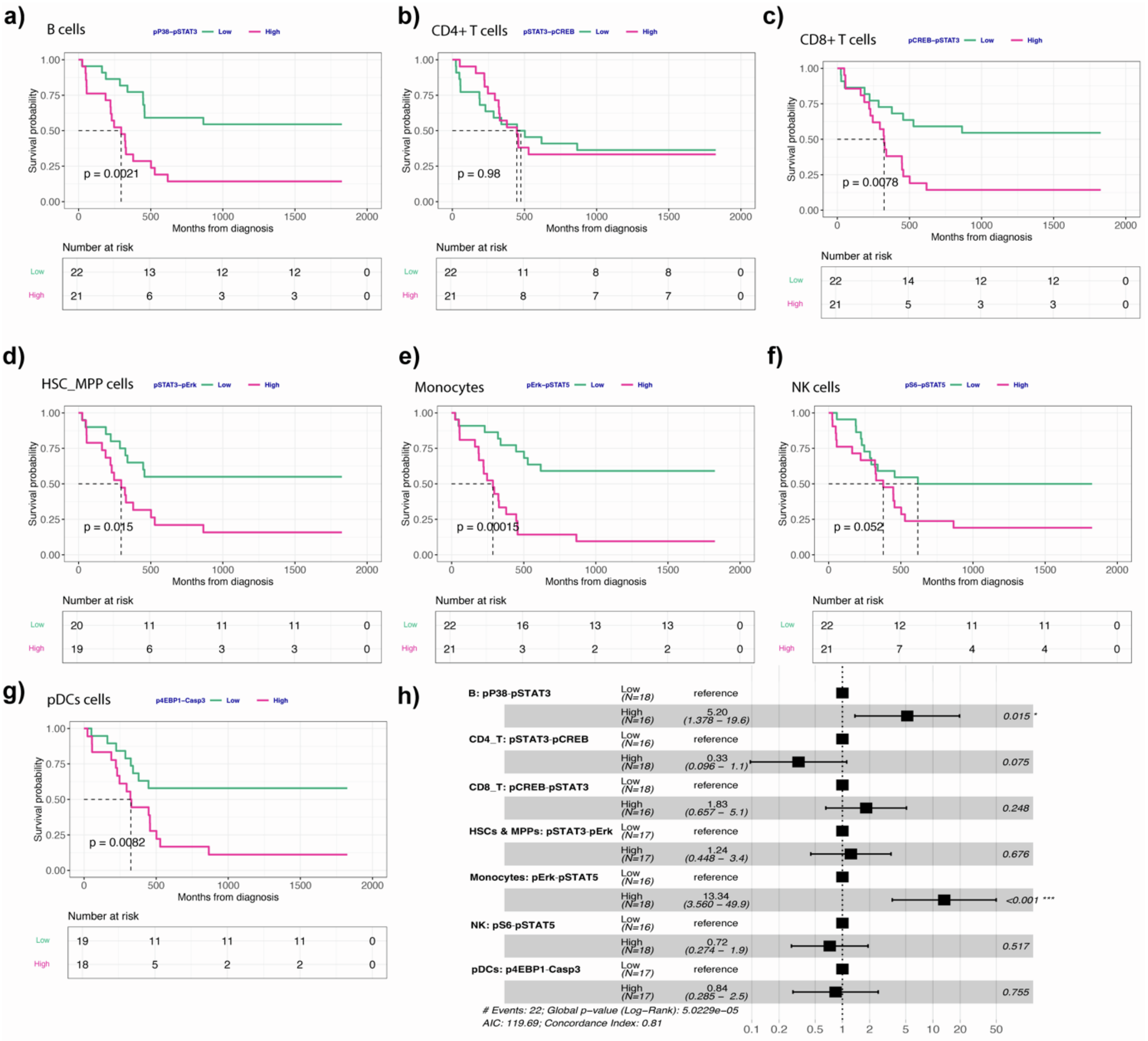

Supplementary Figure 15: Optimising the internal parameters of the classification algorithms used for survival prediction, related to STAR Methods. a-b) Snapshot of the optimisation process for XGBoost's parameter maximum depth of trees; c-d) Snapshot of the optimisation process for LASSO's parameter lamda; e-f) Snapshot of the optimisation process for Ridge regression's parameter lamda. In all cases CD4_T and CD8_T cell type modelling is shown.

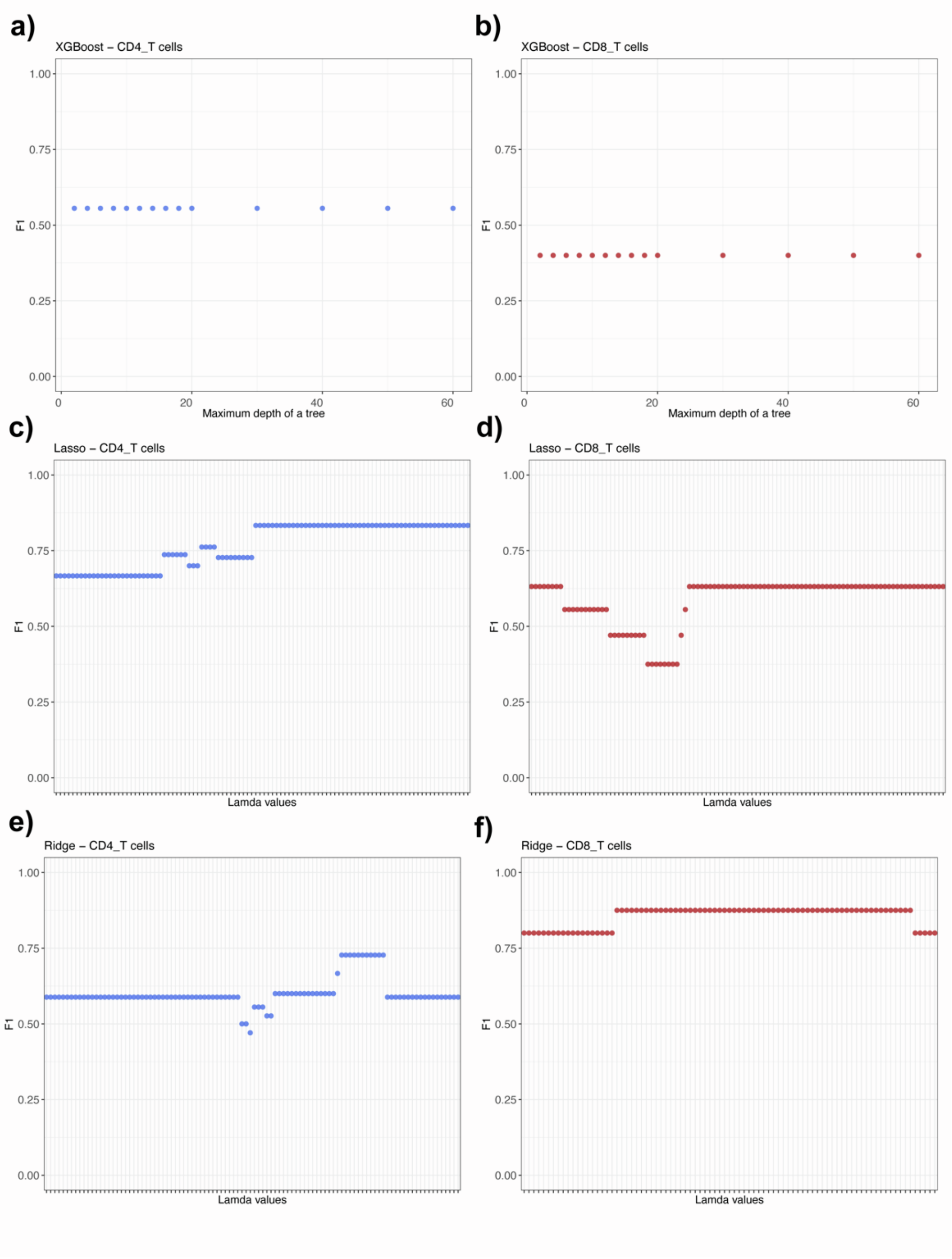

Supplementary Tables

Supplementary Table 1: Characteristics of leukemia patients used in the case study, related to STAR Methods.

Patient ID	original patient nr from ref. 21	Age	5-year survival (days)
P1	P1	35-60	Alive
P2	P2	>60	Alive
P3	P3	<35	Alive
P4	P4	>60	456
P5	P5	35-60	Alive
P6	P6	>60	Alive
P7	P7	>60	246
P8	P8	35-60	189
P9	P9	<35	286
P10	P10	>60	Alive
P11	P11	>60	328
P12	P12	>60	502
P13	P13	>60	Alive
P14	P14	35-60	448
P15	P15	>60	378
P16	P16	>60	221
P17	P17	>60	56
P18	P18	35-60	Alive
P19	P19	>60	51
P20	P20	35-60	Alive
P21	P21	35-60	24
P22	P22	35-60	324
P23	P23	>60	447
P24	P25	>60	229
P25	P26	>60	162
P26	P27	>60	321
P27	P28	35-60	48
P28	P29	>60	Alive
P29	P30	>60	Alive
P30	P31	35-60	Alive
P31	P33	35-60	457
P32	P34	35-60	528
P33	not included	>60	223
P34	not included	>60	295
P35	not included	35-60	339
P36	not included	>60	187
P37	not included	>60	55

P38	not included	35-60	26
P39	not included	35-60	Alive
P40	not included	35-60	Alive
P41	not included	35-60	Alive
P42	not included	35-60	618
P43	not included	35-60	865

Supplementary Table 2: Cohort summary statistics, related to STAR Methods.

	Characteristic	Cohort (n=43)
	Age (average)	55.7
**Sex**	Male	28
	Female	15
**Survival**	Short-term-survivors (STS)	28
	Long-term-survivors (LTS)	15
**FLT3-ITD**	Mutated	7
	Wild type	36
**Karyotype**	Normal	16
	Complex	6
	Chromosomal alter.	17
**Leukemia subtype**	AML	37
	BALL	3
	APL	2
	MDS	1

Abbreviations:
AML: Acute Myeloid Leukemia
BALL: B-cell acute lymphoblastic leukemia
APL: Acute promyelocytic leukemia
MDS: Myelodysplastic syndrome
